# Cytokine Responses to *Schistosoma mansoni* and *Schistosoma haematobium* in Relation to Infection in a Co-endemic Focus in Northern Senegal

**DOI:** 10.1371/journal.pntd.0003080

**Published:** 2014-08-07

**Authors:** Lynn Meurs, Moustapha Mbow, Nele Boon, Kim Vereecken, Abena Serwaa Amoah, Lucja A. Labuda, Tandakha Ndiaye Dièye, Souleymane Mboup, Maria Yazdanbakhsh, Katja Polman

**Affiliations:** 1 Department of Biomedical Sciences, Institute of Tropical Medicine, Antwerp, Belgium; 2 Laboratory of Bacteriology and Virology, Aristide Le Dantec Teaching Hospital, Dakar, Senegal; 3 Department of Parasitology, Leiden University Medical Center, Leiden, The Netherlands; 4 Laboratory of Biodiversity and Evolutionary Genomics, University of Leuven, Leuven, Belgium; 5 Department of Parasitology, Noguchi Memorial Institute for Medical Research, University of Ghana, Legon, Accra, Ghana; 6 Centre de Recherches Médicales de Lambaréné (CERMEL), Lambaréné, Gabon; 7 Institute of Tropical Medicine, University of Tübingen, Tübingen, Germany; University of Nottingham, United Kingdom

## Abstract

**Background:**

In Africa, many areas are co-endemic for the two major *Schistosoma* species, *S. mansoni* and *S. haematobium*. Epidemiological studies have suggested that host immunological factors may play an important role in co-endemic areas. As yet, little is known about differences in host immune responses and possible immunological interactions between *S. mansoni* and *S. haematobium* in humans. The aim of this study was to analyze host cytokine responses to antigens from either species in a population from a co-endemic focus, and relate these to *S. mansoni* and *S. haematobium* infection.

**Methodology:**

Whole blood cytokine responses were investigated in a population in the north of Senegal (n = 200). Blood was stimulated for 72 h with schistosomal egg and adult worm antigens of either *Schistosoma* species. IL-10, IL-5, IFN-γ, TNF-α, and IL-2 production was determined in culture supernatants. A multivariate (i.e. multi-response) approach was used to allow a joint analysis of all cytokines in relation to *Schistosoma* infection.

**Principal Findings:**

*Schistosoma haematobium* egg and worm antigens induced higher cytokine production, suggesting that *S. haematobium* may be more immunogenic than *S. mansoni*. However, both infections were strongly associated with similar, modified Th2 cytokine profiles.

**Conclusions/Significance:**

This study is the first to compare *S. mansoni* and *S. haematobium* cytokine responses in one population residing in a co-endemic area. These findings are in line with previous epidemiological studies that also suggested *S. haematobium* egg and worm stages to be more immunogenic than those of *S. mansoni*.

## Author Summary

In the developing world, over 207 million people are infected with blood-dwelling parasitic *Schistosoma* worms. *Schistosoma haematobium* and *S. mansoni* are the most widespread species. In Africa, they often occur together in the same area, with many people carrying both species. Yet, little is known about the differences in immune response that the human host develops against these two species. It is also unknown whether the presence of one species may affect the immune response to the other. We here investigated 200 people from an area in the north of Senegal where both species occur. They were examined for *Schistosoma* infections, as well as for immune responses to the two species. We observed that both infections were characterized by very similar cytokine responses. However, *S. haematobium* antigens induced higher levels of cytokines than *S. mansoni*. This suggests that *S. haematobium* may give rise to stronger immune responses, and may help to explain differences between the two most important *Schistosoma* species regarding the occurrence of infection and morbidity.

## Introduction

Schistosomiasis is a parasitic disease of major public health importance. *Schistosoma mansoni* and *S. haematobium* are the main human species. Both species are endemic in Africa, where their distributions show a great overlap [1]. Schistosomes are known to down-regulate host immune responses and to induce so-called modified Th2 responses. The exact phenotype of the induced response depends on a complex immunological ‘dialogue’ that involves cytokines and immune cells of Th2, but also Th1, Th17 and regulatory components of the immune system [2].

So far, little is known about differences in host immune responses to schistosomes and possible immunological interactions between *S. mansoni* and *S. haematobium* in humans. Yet, epidemiological studies have suggested that host immunological factors may play an important role in co-endemic areas. Interspecies differences in immunogenicity for example, may explain why infection-age curves and morbidity patterns differ between *S. mansoni* and *S. haematobium*. Also, immunological interspecies differences and/or immunological interactions between *S. mansoni* and *S. haematobium* may explain differences in morbidity levels between single and mixed *Schistosoma* infections. Cheever et al. reported a more pronounced reduction of *S. haematobium* than *S. mansoni* worm loads with age [3]. Similarly, in a mixed focus in northern Senegal, we found the age-infection curve of *S. haematobium* to decline more steeply after adolescence than that of *S. mansoni* [4], indicating that protective immunity against *S. haematobium* may develop more rapidly. In addition, we found that mixed *S. mansoni* and *S. haematobium* infection as compared with single *S. haematobium* infection tended to decrease the risk of *S. haematobium*-specific urinary tract pathology [5]. This appeared mainly due to ectopically excreted, possible hybrid eggs [6]. Others also found *S. mansoni* to affect *S. haematobium*-specific morbidity and vice versa [7], [8], indicating that the two infections may have different effects on the egg-induced immune responses that provoke morbidity.

The present study set out to compare *Schistosoma*-specific cytokine responses induced by *S. mansoni* and *S. haematobium* antigens, and to relate these to *Schistosoma* infection in a *S. mansoni* and *S. haematobium* co-endemic area. *Schistosoma* infection status (single and mixed) and infection intensities as well as *Schistosoma*-specific cytokine responses were determined in residents from a co-endemic focus in northern Senegal. A multivariate (i.e. multi-response) approach was used to allow a joint analysis of multiple cytokine responses (interleukin (IL)-10, IL-5, interferon (IFN)-γ, tumor necrosis factor (TNF)-α, and IL-2) [9].

## Materials and Methods

### Ethics statement

This study was part of a larger investigation on the epidemiology of schistosomiasis and innate immune responses (SCHISTOINIR) for which approval was obtained from the review board of the Institute of Tropical Medicine, the ethical committee of the Antwerp University Hospital and ‘Le Comité National d'Ethique de la Recherche en Santé’ in Dakar. Informed and written consent was obtained from all participants prior to inclusion into the study. For minors, informed and written consent was obtained from their legal guardians.

All community members were offered praziquantel (40 mg/kg) and mebendazole (500 mg) treatment after the study according to WHO guidelines [10].

### Study area

This study was conducted in Ndieumeul and Diokhor Tack, two neighboring communities on the Nouk Pomo peninsula in Lake Guiers. Details on the study area have been described elsewhere [4], [5]. Between July 2009 and March 2010, parasitological data were collected from 857 individuals [4]. A random subsample of 200 subjects was followed up immunologically. These subjects were between 5 and 53 years of age. Individuals who had lived in an urban area in the 5 years preceding the study (n = 7), had taken praziquantel within the last year (n = 2), or had clinical signs of malaria (recruited upon recovery), and pregnant women (n = 18) were excluded from the immunological study.

### Parasitology

Two feces and two urine samples were collected from each participant on consecutive days. Infection with *Schistosoma* spp. was determined quantitatively (by Kato-Katz and urine filtration), and infection with soil-transmitted helminths (STHs) *Ascaris lumbricoides*, *Trichuris trichiura* and hookworm, was assessed qualitatively (by Kato-Katz), as described elsewhere [4]. Aliquots of the first fecal samples were preserved in ethanol to confirm microscopy results by multiplex PCR (*A. lumbricoides*, hookworm and *Strongyloides stercoralis*) (n = 198) [11]. Infection with *Plasmodium* was determined by Giemsa-stained thick blood smears.

### Whole blood culture

Five hours after venipuncture, heparinized blood was diluted 1∶4 in RPMI 1640 (Invitrogen) supplemented with 100 U/ml penicillin, 100 µg/ml streptomycin, 1 mM pyruvate and 2 mM glutamate (all from Sigma). This mixture (200 µl sample volume) was incubated in 96-well round bottom plates (Nunc) at 37°C under 5% CO_2_ atmosphere for 72 h, together with one of four schistosomal water-soluble antigen preparations at a final concentration of 10 µg protein/ml:

1. *Schistosoma* egg antigen (SEA) derived from *S. mansoni* (SEAm);
2. SEA from *S. haematobium* (SEAh);
3. Adult worm antigen (AWA) from *S. mansoni* (AWAm); or
4. AWA from *S. haematobium* (AWAh).

Medium (see above) without stimulus was used as a negative control. After harvesting, supernatants were stored at −80°C. *Schistosoma* eggs and adult worms were isolated from either *S. mansoni*- or *S. haematobium*-infected golden hamsters. SEAm, SEAh, AWAm and AWAh were prepared from this material using identical procedures. In brief, eggs or worms were freeze-dried and then homogenized in phosphate-buffered saline (PBS) with 10% n-octyl-β-D-glucopyranoside. Subsequently, this mixture was sonicated, frozen, thawed and washed with PBS. The resulting pellet was dialyzed and filter-sterilized. While AWAm and AWAh batches were lipopolysaccharide (LPS)-free, SEAm and SEAh antigens contained equivalent amounts of LPS (final concentrations of 1–5 ng/ml).

### Cytokine measurement

IL-10, IL-5, IFN-γ, TNF-α, and IL-2 in culture supernatants were analyzed simultaneously using custom Luminex cytokine kits (Invitrogen) according to the manufacturer's instructions. Samples with concentrations below the detection limit were assigned values corresponding to half of the lowest value detected. Lowest values detected were 0.063 pg/ml for IL-10, 0.044 pg/ml for IL-5, 0.090 pg/ml for IFN-γ, 0.051 pg/ml for TNF-α, and 0.063 pg/ml for IL-2.

### Statistical analysis

Results were considered significant when the *p*-value was <0.05. The Pearson Chi-square test was used to determine the association between infection status on the one hand, and age and gender on the other. Nonparametric techniques were chosen because cytokine concentrations were not normally distributed. Univariate statistics were used to compare single antigen-induced responses within individuals (IBM SPSS 21.0). McNemar's tests were used to compare cytokine response frequencies between *S. mansoni* and *S. haematobium* antigen-induced responses within individuals (e.g. SEAm- versus SEAh-induced responses). Similarly, Wilcoxon Signed Rank tests were used to compare cytokine response levels between *S. mansoni* and *S. haematobium* antigen-induced responses within individuals. Multivariate (i.e. multi-response) statistics were used to collectively analyze multiple cytokine responses – i.e. cytokine profiles - in the study population, and to investigate interrelationships between these responses [9]. We chose the nonparametric technique nonmetric multidimensional scaling (nMDS; in R with the ‘Vegan’ package [12], [13]). This is a variant of the parametric principal component analysis (PCA), but with fewer assumptions about the nature of the data and the interrelationship of the variables [14]. This is important because cytokine response levels were not normally distributed, even after log-transformation. Also, levels of different cytokines typically correlate with one another. Upon computation of the cytokine profiles, associations between these cytokine profiles and *Schistosoma* infection were assessed. The approach is illustrated in Supporting Information S1. Before nMDS, cytokine concentrations in the negative control were subtracted from those in antigen-stimulated samples to obtain net cytokine responses. Negative values were set to zero. Net cytokine responses were normalized by log(base 10)-transformation after adding 1 pg/ml to allow for zeroes. *Schistosoma* infection intensities were normalized after adding half of the detection limit (i.e. 5 eggs per gram of feces and 0.5 eggs per 10 ml of urine for *S. mansoni* and *S. haematobium*, respectively). One nMDS was performed for each of the four *Schistosoma*-specific whole blood stimulations (either SEAm, SEAh, AWAm or AWAh) using the ‘metaMDS’ function [13]. Each nMDS was repeated several times to assess the robustness of the resulting pattern [14]. The Euclidean dissimilarity index was used [13], and cytokine profiles - i.e. the matrix of IL-10, IL-5, IFN-γ, TNF-α, and IL-2 - were plotted in three dimensions (3D) to adequately represent the variation in the data [14]. Afterwards, gradients of the separate cytokine responses, on which the nMDS was based, were fitted using the ‘envfit’ function [13]. The same function was used to fit infection intensities onto each 3D nMDS, and to statistically test associations of antigen-induced cytokine profiles with *Schistosoma* infection intensity or infection status, i.e. uninfected, single *S. mansoni*, single *S. haematobium*, versus mixed *S. mansoni* and *S. haematobium* infection. The ‘ordiellipse’ function was used to fit average group scores - with their 95% confidence intervals (CIs) - for different infection statuses [13]. In contrast to individual *S. mansoni*- and *S. haematobium*-induced cytokine responses which can be compared quantitatively within individuals as described above (univariate statistics), qualitative differences between *S. mansoni*- and *S. haematobium*-induced cytokine profiles could only be assessed visually by nMDS, not by formal statistical testing.

## Results

### Characteristics of the study population

The study population consisted of 88 males and 112 females with a median age of 16 (range 5–53) years. Malaria and STHs *T. trichiura* and hookworm were absent in this population, and *A. lumbricoides* and *S. stercoralis* rare (n = 3 and 2, respectively, with 100% concordance between microscopy and PCR). In contrast, 137 (69%) subjects were infected with *S. mansoni*, and 116 (58%) with *S. haematobium*. Sixty percent (95/158) of all *Schistosoma* infections were mixed *S. mansoni* and *S. haematobium* infections (Table 1). The distributions of *S. mansoni* and *S. haematobium* infections in the study population according to age and gender are shown in Table 2. Both *Schistosoma* infections peaked in adolescents (10 to 19 year-olds), but gender differences were not statistically significant. Epidemiological patterns of infection have been described in more detail elsewhere [4].

**Table 1:** *Schistosoma* infections in the study population.

	*S. mansoni* infection		*S. haematobium* infection		Prevalence (n)	Code for Infection Status In Figure 2
Subjects	Feces	Urine^a^	Feces	Urine		
**Positive**					**158**	
Single infections					63	
	+	−	−	−	42	M (dark blue)
	−	−	−	+	21	H (light blue)
Mixed infections					95	MH
	+	−	−	+	81	MH (pink)
	+	+	−	+	13	MH (yellow)
	−	+	−	+	1	MH (red)
**Negative**	−	−	−	−	**42**	N (green)
**Total**	**136**	**14**	**0**	**116**	**200**	

a *Schistosoma mansoni* eggs that were ectopically excreted in the urine had a *S. mansoni*-like morphology but may have had a genetically hybrid constitution [4], [6].

**Table 2:** Distribution of *Schistosoma* infection in the study population.

	n	*S. mansoni* infection		*S. haematobium* infection	
		Percentage of positives	*p*-value	Percentage of positives	*p*-value
**Age (in years)**			0.001		0.001
5–9	51	58.8		66.7	
10–19	59	88.1		72.9	
20–39	55	58.2		49.1	
≥40	35	65.7		34.3	
**Gender**			0.32		0.20
Male	88	72.7		63.6	
Female	112	65.2		53.6	

### General cytokine profiles

**Figure 1:**
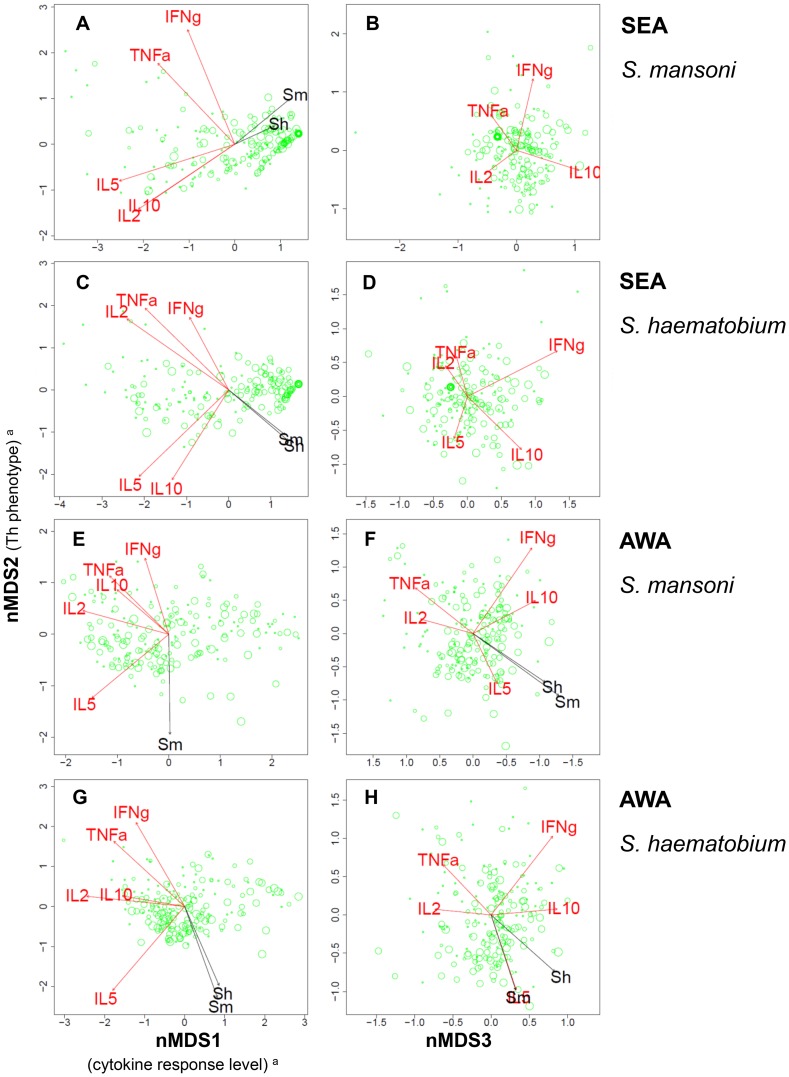
Variation in *Schistosoma* antigen-induced cytokine responses in relation to *Schistosoma* infection intensity. Each three-dimensional (3D) nMDS ordination is represented in two 2D planes (Supporting Information S1). Left and right panels represent the 1^st^ and 2^nd^, and 2^nd^ and 3^rd^ dimensions, respectively. **Panels A** and **B** show the *S. mansoni* egg antigen (SEAm)-induced cytokine profile, **Panels C** and **D** that of *S. haematobium* SEA(h), **Panels E** and **F** that of *S. mansoni* adult worm antigens (AWAm), and **Panels G** and **H** show *S. haematobium* AWA(h)-induced cytokine profiles. Green dots represent individuals. Distances between dots approximate the rank order of dissimilarities in cytokine profiles between the respective individuals with stress values (i.e. discrepancies) of 0.051 for SEAm, 0.041 for SEAh, 0.058 for AWAm, and 0.061 for AWAh. Red arrows indicate linear gradients of normalized net cytokine responses on which the nMDS is based. Green dot sizes are proportional to individual values of normalized infection intensity of *S. mansoni* (for simplicity dots were only labelled with *S. mansoni* (not *S. haematobium*) infection intensity). Black arrows indicate linear gradients of post hoc fitted normalized infection intensity of *S. mansoni* (‘Sm’) and *S. haematobium* (‘Sh’). The length of the arrows is proportional to the goodness of fit onto the cytokine profile within one 2D plane, but lengths cannot be compared between cytokine and infection intensity arrows. Arrows are only depicted if their fit was significant at the level of *p* = 0.05 in 3D ordinations (see Table 4), as well as in the respective 2D planes. In Panel H, the arrows of IL-5 response and *S. mansoni* infection intensity are overlapping and their labels are therefore illegible. ^a^The biological a posteriori interpretation of nMDS1 (left x-axis) and nMDS2 (y-axis) were added between brackets on the axis labels, but nMDS3 (right x-axis) could not be interpreted.

**Figure 2:**
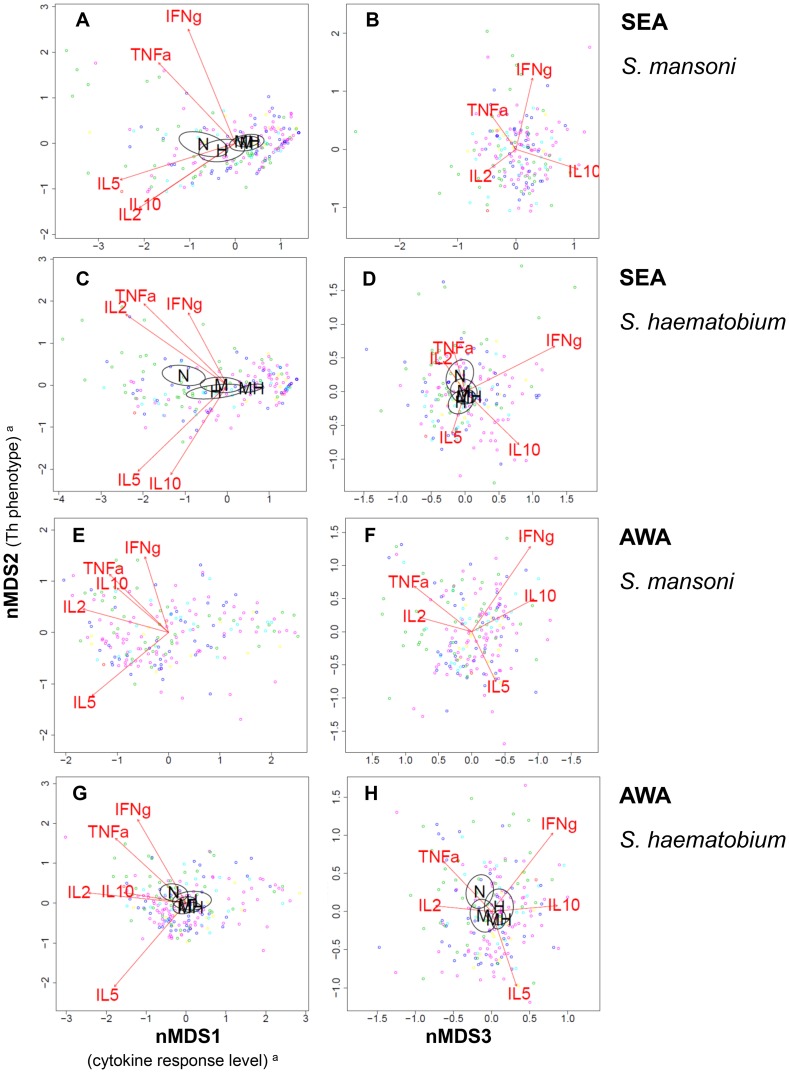
Variation in *Schistosoma* antigen-induced cytokine responses in relation to *Schistosoma* infection status. Each three-dimensional (3D) nMDS ordination is represented in two 2D planes (Supporting Information S1) as in Figure 1: Left and right panels represent the 1^st^ and 2^nd^, and 2^nd^ and 3^rd^ dimensions, respectively. **Panels A** and **B** show the *S. mansoni* egg antigen (SEAm)-induced cytokine profile, **Panels C** and **D** that of *S. haematobium* SEA(h), **Panels E** and **F** that of *S. mansoni* adult worm antigens (AWAm), and **Panels G** and **H** show *S. haematobium* AWA(h)-induced cytokine profiles. Dots represent individuals and distances between dots approximate the rank order of dissimilarities in cytokine profiles between the respective individuals with stress values (i.e. discrepancies) of 0.051 for SEAm, 0.041 for SEAh, 0.058 for AWAm, and 0.061 for AWAh. Red arrows indicate linear gradients of normalized net cytokine responses on which the nMDS is based. The length of the arrows is proportional to the goodness of fit onto the cytokine profile within one 2D plane, and arrows are only depicted if their fit was significant at the level of *p* = 0.05 in 3D ordinations (see Table 4), as well as in the respective 2D planes. Green dots represent uninfected individuals, dark blue those with single *S. mansoni* infections, light blue single *S. haematobium*, and the other colors indicate people with mixed infections: pink indicates mixed infections without ectopic egg elimination, yellow mixed infections with *S. mansoni* in feces as well as in urine and *S. haematobium* in urine, and red dots represent one individual with both *S. mansoni* and *S. haematobium* eggs in urine (possibly a hybrid species [4]–[6]; see also Table 1). Ellipsoids represent 95% confidence intervals for average group scores, for different infection statuses: uninfected (‘N’), single *S. mansoni* (‘M’), single *S. haematobium* (‘H’), versus mixed infection (‘MH’). Ellipsoids are drawn using the function ‘ordiellipse’, and only depicted if the fit of infection status onto the cytokine profile was significant at the level of *p* = 0.05 in 3D ordinations (see Table 4), as well as in the respective 2D planes. In Panel A and G, the labels for single *S. mansoni* (‘M’) and mixed infection (‘MH’) are overlapping. ^a^The biological a posteriori interpretation of nMDS1 (left x-axis) and nMDS2 (y-axis) were added between brackets on the axis labels, but nMDS3 (right x-axis) could not be interpreted.

Insight into the different antigen-induced cytokine responses relative to one another was obtained by nMDS. Figure 1 and 2 show the variation in multivariate cytokine responses in the study population, with dots representing individuals. Distances between dots approximate inter-individual dissimilarities in cytokine responses with stress values (i.e. discrepancies) of 0.051 for SEAm, 0.041 for SEAh, 0.058 for AWAm, and 0.061 for AWAh. Red arrows indicate increasing gradients of IL-10, IL-5, IFN-γ, TNF-α and IL-2 responses, respectively. The level of a cytokine response increases in the direction of the corresponding arrow (see also Supporting Information S1). The length of a cytokine arrow indicates the goodness of fit of that arrow (or cytokine gradient).

The nMDS outcomes for the first axis (nMDS1) show that for each of the four antigen stimulations, all cytokine responses point to the left. Individuals plotted on the left produced consistently higher levels of all cytokines measured than those on the right. In other words, nMDS1 indicates a gradient of high (left) to low (right) cytokine responses. In analogy, the second axis (nMDS2), indicates a gradient of Th1-like (IFN-γ and TNF-α, top) to Th2-like (IL-5, bottom) phenotypes for each of the antigen stimulations. In contrast to SEA-induced IL-5, AWA-induced IL-5 was not accompanied by production of IL-10. IL-2 levels increased with Th1 cytokines, except for SEAm. The third axis (nMDS3) indicates a gradient of TNF-α and IL-2 (left) to IFN-γ and IL-10 (right).

In contrast to antigen-induced cytokines, spontaneously induced levels of cytokines in the control (medium only), did not show significant gradients, except for IL-5 on the third nMDS axis (stress = 0.11, data not shown).

### Comparison between *S. mansoni*- and *S. haematobium*-induced cytokine responses and cytokine profiles

Figure 1 and 2 indicate that *S. mansoni* and *S. haematobium* antigens induced very similar cytokine profiles; cytokine profiles differed more between adult (AWA) and egg (SEA) life stages of the parasite than between the two *Schistosoma* species. Within individuals, *S. haematobium*-induced cytokine response levels were higher than those induced by *S. mansoni* (Table 3). This was statistically significant for all SEA- and AWA-induced cytokine responses that were measured, except for SEA-induced IFN-γ and IL-10.

**Table 3:** Levels of *Schistosoma*-induced cytokine responses in 72 h whole blood cultures (n = 200).

Antigen	Species	Cytokine	Response (%)	Median Concentration in pg/ml (IQR)^a^		*p*-value^b^
**SEA** c	***S. mansoni***					
		IL-10	92.0	12.7	(5.2–32.4)	0.874
		IL-5	78.5	3.7	(1.0–19.0)	**<0.001**
		IFN-γ	67.5	3.4	(0.05–7.8)	0.729
		TNF-α	64.5	0.7	(0.03–2.2)	**0.046**
		IL-2	80.0	6.3	(2.0–18.8)	**<0.001**
	***S. haematobium***					
		IL-10	90.5	13.1	(4.7–32.2)	
		IL-5	77.0	5.2	(0.9–47.4)	
		IFN-γ	63.0	4.2	(0.05–7.8)	
		TNF-α	67.5	1.0	(0.03–4.3)	
		IL-2	80.5	8.2	(2.1–54.7)	
**AWA** d	***S. mansoni***					
		IL-10	98.5	25.7	(13.2–48.2)	**0.008**
		IL-5	94.5	69.3	(11.8–201.2)	**<0.001**
		IFN-γ	74.5	5.4	(0.05–9.4)	**0.002**
		TNF-α	90.5	4.6	(1.2–10.9)	**<0.001**
		IL-2	98.0	60.3	(22.4–152.1)	**<0.001**
	***S. haematobium***					
		IL-10	99.0	30.0	(17.0–50.4)	
		IL-5	96.0	108.6	(25.9–237.9)	
		IFN-γ	78.5	6.3	(1.7–12.1)	
		TNF-α	96.5	6.0	(2.7–15.1)	
		IL-2	98.0	99.5	(42.4–224.5)	
**None**						
		IL-10	59.5	1.7	(0.03–4.9)	
		IL-5	57.0	0.9	(0.02–2.6)	
		IFN-γ	58.0	2.2	(0.05–5.8)	
		TNF-α	63.5	0.4	(0.03–1.5)	
		IL-2	45.5	0.03	(0.03–2.9)	

Blood samples from one individual were divided into five and stimulated with *Schistosoma* antigens (SEAm, SEAh, AWAm, or AWAh), and with medium only (negative control; see Materials and Methods).

a Crude cytokine levels are reported. IQR: Interquartile range (Tukey's hinges).

b Wilcoxon Signed Rank test comparing *S. mansoni*- and *S. haematobium*-induced cytokine levels within individuals (either for SEA or AWA).

c *Schistosoma* egg antigen.

d Adult worm antigen.

### Relation between cytokine profiles and *Schistosoma* infection intensity

Subsequently, we related the above-described variation in cytokine responses in the study population (i.e. plotted cytokine profiles) to infection intensity. Table 4 shows that all associations between *Schistosoma* antigen-induced cytokine profiles and infection intensity were statistically significant. In Figure 1, the direction of the black arrows represents the increasing gradients of *S. mansoni* and *S. haematobium* infection intensity, respectively (see also Supporting Information S1). On the first axis, which indicates cytokine response levels (see above), these arrows generally point into the opposite direction of cytokine responses. This indicates that people with elevated *Schistosoma* infection intensities are more likely to have lower cytokine responses, and vice versa. On the second axis, which indicates the Th1 versus Th2 response phenotype (see above), infection intensity generally increases with IL-5 and decreases with Th1 cytokines TNF-α, IFN-γ, and IL-2 (except for SEAm-induced IL-5 which decreases with increasing infection intensity). Briefly, as infection intensity increased, cytokine response levels decreased and the Th2 phenotype became more pronounced. The association between infection intensity and reduced cytokine responsiveness was more pronounced for SEA than for AWA stimulation. *Schistosoma* infection intensity increased with AWA-induced IL-5, but decreased with SEA-induced IL-5 levels, indicating that people with higher infection intensities produced more of a Th2-like response against AWA and more of a suppressive response (i.e. with low cytokine response levels) against SEA than people with lower infection intensities, and vice versa.

**Table 4:** Association between *Schistosoma* infection and *Schistosoma* antigen-induced cytokine profiles.

Infection	Antigen-induced cytokine profile			
	SEAm	SEAh	AWAm	AWAh
***S. mansoni*** ** infection intensity**				
R^2^	0.14	0.17	0.10	0.13
*p*-value	**0.001**	**0.001**	**0.001**	**0.001**
***S. haematobium*** ** infection intensity**				
R^2^	0.05	0.18	0.07	0.15
*p*-value	**0.02**	**0.001**	**0.003**	**0.001**
**Infection Status**				
R^2^	0.09	0.18	0.02	0.04
*p*-value	**0.001**	**0.001**	0.2	**0.01**

Figure 1 shows the fit of infection intensity and Figure 2 that of infection status (uninfected, single *S. mansoni*, single *S. haematobium*, versus mixed infections) onto each of the four *Schistosoma* antigen-induced cytokine profiles (either SEAm, SEAh, AWAm or AWAh), obtained by the ‘metaMDS’ and ‘envfit’ functions (see also Supporting Information S1) [12], [13]. Here, the goodness of these fits, i.e. squared correlation coefficients (R^2^), are shown. The statistical significance was assessed using permutation tests (n = 999), and presented *p*-values are approximations.

We did not observe differences in induced cytokine profiles between the two *Schistosoma* infections. Associations between cytokine profiles and infection intensity were comparable for *S. mansoni* and *S. haematobium* infections (Figure 1). Table 4 shows significant correlations between cytokine profiles and *Schistosoma* infection intensity for homologous combinations (i.e. infection intensity and antigen stimulation of the same species) as well as for heterologous combinations (i.e. infection intensity of one and antigen stimulation of the other species).

### Relation between cytokine profiles and infection status (mixed versus single infections)

*Schistosoma* antigen-induced cytokine profiles were significantly associated with *Schistosoma* infection status, except upon stimulation with AWAm (Table 4). Figure 2 shows how antigen-induced cytokine profiles differed according to infection status (except for AWAm, which was not significantly associated with infection status), with 95% CI ellipsoids indicating the average nMDS scores per infection group: uninfected (‘N’), single *S. mansoni* (‘M’), single *S. haematobium* (‘H’), versus mixed (‘MH’) *Schistosoma* infection group. In analogy with Figure 1, uninfected individuals had higher cytokine responses than *Schistosoma*-infected subjects, and their cytokine profiles were skewed more towards the Th1 phenotype. On the whole, there was a gradient in cytokine profiles from uninfected individuals, to people with single and then mixed *Schistosoma* infections (Figure 2) and these profiles were in the same direction as the gradient of infection intensity (Figure 1). In other words, people with low cytokine responses of the Th2 phenotype tended to have both mixed and heavier infections, people with strong Th1 responses tended to be uninfected, and those with an intermediate cytokine profile tended to have both single and lighter *Schistosoma* infections.

For the SEAm-induced cytokine profile, there was a clear difference (i.e. separation between ellipsoids) between *S. mansoni*-infected individuals (with either single or mixed *S. mansoni*), and those without *S. mansoni* (no *Schistosoma* infection, or single *S. haematobium* infection; Figure 2A). There were no significant differences in this cytokine profile between single and mixed *S. mansoni* infections, or between uninfected individuals and those with single *S. haematobium* infections. This indicates that, in contrast to *S. mansoni*, *S. haematobium* infection status was not associated with SEAm-induced cytokine profiles. *Schistosoma haematobium*-induced cytokine profiles on the other hand, showed similar relationships with *S. mansoni* as well as with *S. haematobium* infection status. Cytokine profiles of people with single and mixed infections differed significantly from those of uninfected people, and cytokine profiles did not appear to differ between single *S. mansoni* and single *S. haematobium* infections.

## Discussion

The objective of this study was to compare cytokine responses induced by *S. mansoni* and *S. haematobium* antigens, and to relate these to *Schistosoma* infection in a *S. mansoni* and *S. haematobium* co-endemic area. We showed that *Schistosoma* infection intensity was significantly associated with *Schistosoma* antigen-induced cytokine profiles and that it may explain up to 18% of the variation in cytokine responses observed in this population. As *Schistosoma* infection intensity increased, cytokine responses decreased and the Th2 phenotype became more pronounced. This was exemplified by relatively higher IL-5 (and IL-10) and relatively lower IFN-γ, TNF-α and IL-2 levels. Lightly infected and uninfected subjects on the other hand, had elevated cytokine responses, with a Th1 phenotype. These patterns are consistent with the modified Th2 response characteristic for schistosomiasis [2]. nMDS also indicated that the association between infection and the Th2 phenotype was more pronounced for AWA, while that between infection and (reduced) cytokine responsiveness was more pronounced for SEA. These observations fit with a previous study by Joseph et al. describing similar immunological differences between *Schistosoma* adult worm and egg life stages in a population from a *S. mansoni* mono-endemic area, using more conventional analyses [15].

Secondly, we demonstrated that increased *Schistosoma* infection intensity and mixed (as compared to single) infections were associated with similar, modified Th2, cytokine profiles. This is probably due to the fact that subjects with mixed infections were more likely to have higher infection intensities than those with single infections [4]. Also, similar, modified Th2, cytokine profiles were observed for both *S. mansoni* and *S. haematobium* infection intensity, whether blood was stimulated with antigens from the homo- or heterologous species. This may be indicative of immunological cross-reactivity between species. For *S. mansoni*-induced cytokine profiles however, this was unlikely, because profiles did not differ between single and mixed *S. mansoni* infection groups. While *S. haematobium*-induced cytokine profiles did differ between single and mixed *S. haematobium* infection groups, we could not determine whether these differences were due to mixed infection per se, or to higher *S. haematobium* infection intensity in mixed as compared to single infections. Other potentially confounding factors such as age may have been involved as well [4], and future studies should be performed to assess their respective roles in determining cytokine responses. To obtain more evidence on the existence of cross-reactivity between the two major human *Schistosoma* species, it is important to compare immune responses between different co- and mono-endemic areas, using different immunological parameters (e.g. cytokine, humoral and cytological data). To our knowledge, only one human study reported on functional *S. mansoni* – *S. haematobium* cross-reactivity. This study from 1974 reported lethal in vitro activity of sera from subjects infected with one species against schistosomula of the same but not of the other species [16]. Indeed, *S. mansoni* and *S. haematobium* may share few if any epitopes that are involved in protective immunity because they belong to genetically distinct groups. Potential cross-reactivity or the lack thereof merits further investigation as this may have important implications for our understanding of the epidemiology of schistosomiasis as well as for the development of an effective schistosomiasis vaccine.

The present study demonstrated that nMDS can be used successfully to analyze host cytokine responses collectively. In this way, it was possible to analyze cytokine responses in relation to one another, and in relation to *Schistosoma* infection. nMDS is a nonparametric, multivariate and visual method. It is a robust and powerful tool because it avoids problems of multiple statistical tests and violations of data assumptions [14]. Moreover, nMDS makes it easier to interpret complex data than traditional one-by-one graphs, tables, and tests. Here, we used this approach to study multivariate cytokine responses, but it can be used equally well to increase our understanding of other complex, multidimensional data, such as cytological and/or serological data (Durnez et al, unpublished data), as well as infection data on multiple co-endemic parasite species.

Additional analyses showed that, within individuals, *S. haematobium* antigens induced higher cytokine responses in 72 h whole blood cultures than those of *S. mansoni*. A very similar pattern was observed in parallel investigations in Ghana, in a population which was - in contrast to the Senegalese study population - first exposed to *S. haematobium* and then to both *S. mansoni* and *S. haematobium*, and with lower prevalences of *S. mansoni* and higher prevalences of *S. haematobium* (unpublished data, A.S. Amoah et al, and ref [4]). This suggests that this finding does not depend on the level of transmission or on exposure history, and that the two *Schistosoma* species may differ in their immunogenicity. This hypothesis is in line with observations from Van Remoortere et al. who found *S. mansoni* to induce mainly IgM antibodies – which are thought to inhibit protective host immune responses [17] – while *S. haematobium* induced both IgM and IgG antibodies against shared carbohydrate epitopes [18]. It is therefore tempting to speculate that lower cytokine response levels may prevent Ig class switching from IgM to IgG for these epitopes in *S. mansoni* infection, while stronger cytokine responses may promote class switching in *S. haematobium* infection. Alternatively, differences in their biochemical composition may underlie interspecies differences in both immunogenicity and humoral immune responses. These two immunological interspecies differences may also have contributed to earlier epidemiological findings. Several studies observed a steeper decline of the age-infection curve of *S. haematobium* as compared to *S. mansoni* after adolescence, indicating that protective immunity against *S. haematobium* might develop more rapidly [3], [4]. Secondly, higher levels of *S. haematobium*- as compared to *S. mansoni*-specific morbidity have been observed in co-endemic populations [5], [7], [8], suggesting that the immune responses provoked by *S. haematobium* eggs might be more pathogenic. It should be noted however, that other factors may also explain these two epidemiological observations. For example, *S. mansoni* and *S. haematobium* eggs accumulate in different organs, i.e. the liver and the urinary tract, respectively, and these differences in anatomical context may also explain the differences in the extent of morbidity between the two species. More research is necessary to investigate the abovementioned immunological interspecies differences and their implications for epidemiological patterns of infection and morbidity in more detail.

### Conclusion

In conclusion, this is the first study to comprehensively investigate *S. mansoni*- and *S. haematobium*-induced cytokine responses in a *S. mansoni* and *S. haematobium* co-endemic area, and to relate these cytokine responses to *Schistosoma* infection. The present study demonstrates that nMDS can be used successfully as a tool for the joint analysis of multiple cytokine responses in relation to *Schistosoma* infection. We showed strong associations between *Schistosoma* infection and *Schistosoma*-induced cytokine profiles, and provided a first insight into potential differences and interactions between human *S. mansoni* and *S. haematobium* infections. This knowledge will contribute to an improved understanding of the mechanisms underlying *Schistosoma* infection and morbidity in co-endemic populations.

## Supporting Information

Supporting information S1 **Schematic representation of nonmetric multidimensional scaling.**
(DOCX)
