# Supplementary material for: Cytokine Responses to Schistosoma mansoni and Schistosoma haematobium in Relation to Infection in a Co-endemic Focus in Northern Senegal
Source: PLoS Negl Trop Dis. 2014 Aug 7;8(8):e3080. doi: 10.1371/journal.pntd.0003080 (PMC4125161; doi:10.1371/journal.pntd.0003080)
Supplement: Supporting information S1 — Schematic representation of nonmetric multidimensional scaling. (DOCX) [file pntd.0003080.s001.docx]

**Supporting information S1: Schematic representation of nonmetric multidimensional scaling.**


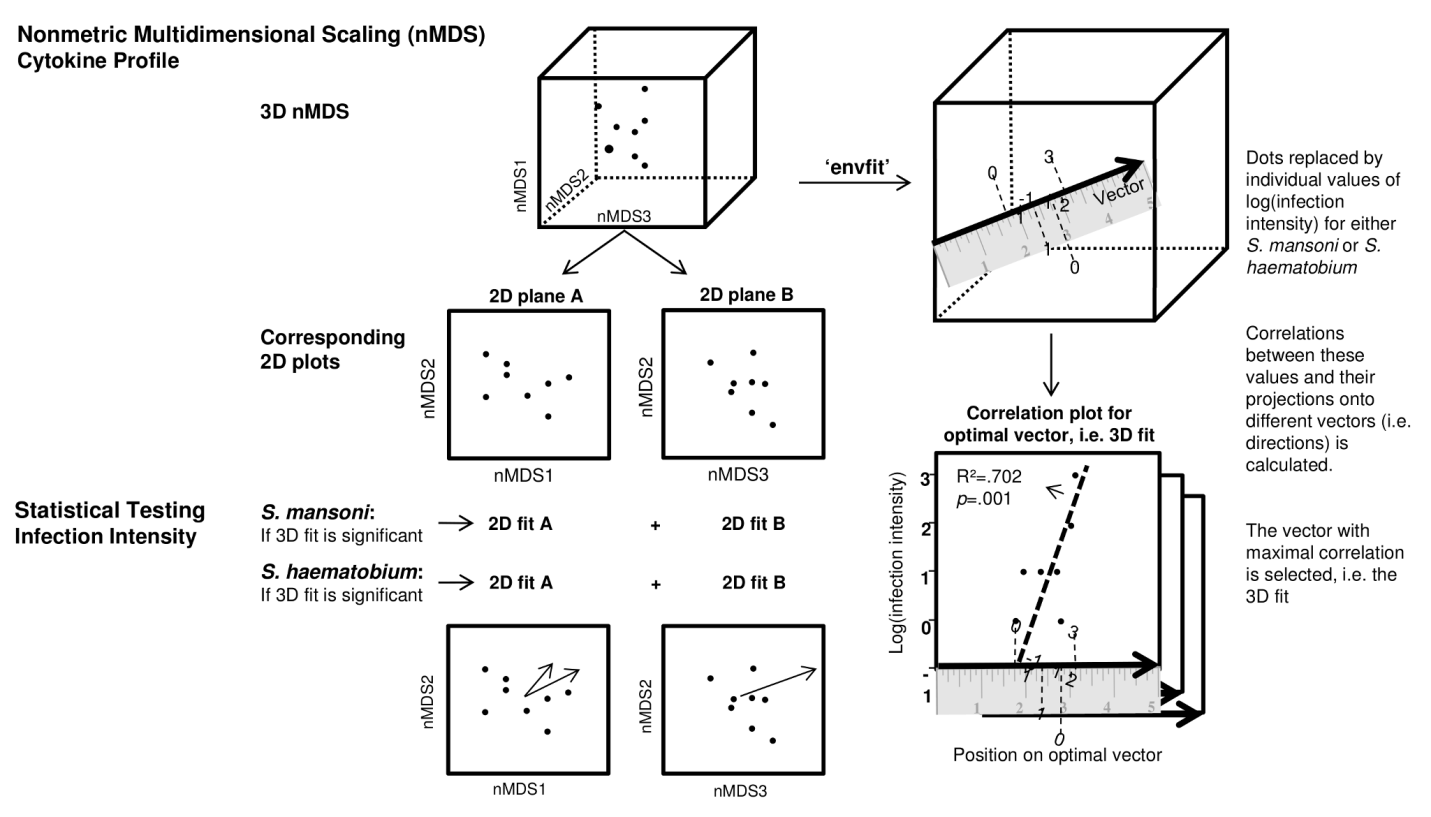
The nonparametric data reduction technique nonmetric multidimensional scaling (nMDS) aims to plot the individuals (dots) in such a way that the inter-point distance in the plot approximates the same rank order as corresponding dissimilarities in multivariate cytokine profiles (i.e. matrix of net IL-10, IL-5, IFN-γ, TNF-α, and IL-2 production) [9,12–14]. In other words: it aims to minimize the stress, i.e. discrepancy between inter-point distances and true dissimilarities. This is achieved in an iterative procedure. Subsequently, the plot (i.e. a 3D cloud of dots) is rotated in such a way that the first axis represents the largest variance, followed by gradually less variance in the consecutive axes. This method is analogous to principal component analysis which is, in fact, the parametric equivalent of nMDS. In order to obtain acceptable stress levels and robust patterns, it was necessary to plot individuals in three dimensions (3D)[14]. One 3D nMDS (cube) was performed for each of the four *Schistosoma*-specific whole blood stimulations (either SEAm, SEAh, AWAm or AWAh) using the ‘metaMDS’ function (in R using the ‘Vegan’ package [12,13]). Afterwards, the ‘envfit’ function was used to test whether a gradient of infection intensity is present in the 3D cloud of dots. This is done by searching the vector (direction represented by an arrow) that shows a maximal correlation with the individual values of log-transformed infection intensity [13]. *Schistosoma mansoni* as well as *S. haematobium* infection intensity were fitted onto each 3D cytokine profile. Table 4 presents the goodness of fit (R²) and statistical significance (*p*-value) for each of these combinations. Two 2D planes (squares) were produced from each 3D nMDS: A) nMDS axis 1 by 2; and B) axis 3 by 2. Significant 3D correlations were tested and fitted (pointers) in the two 2D planes (Figure 1). Similarly, ‘envfit’ was used to test whether people with different infection status (uninfected, single *S. mansoni*, single *S. haematobium*, versus mixed infections) differed in their cytokine profiles (not shown). The ‘ordiellipse’ function was used to visualize how cytokine responses varied with infection status (Figure 2).
